# Microbial biofilms promote biomineralization and reduce leaf decay under controlled ionic conditions

**DOI:** 10.1016/j.bioflm.2026.100403

**Published:** 2026-10-02

**Authors:** Sabina Karačić, Brianne Palmer, Jesenko Karačić, Fabian Gäb, Esther Sib, Carole T. Gee, Gabriele Bierbaum

**Affiliations:** a Institute of Medical Microbiology, Immunology and Parasitology, University Hospital Bonn, Venusberg‐Campus 1, Bonn, 53127, Germany; b Bonn Institute of Organismic Biology, Division of Paleontology, University of Bonn, Bonn, 53115, Germany; c Institute of Geosciences, University of Bonn, Bonn, 53115, Germany; d Institute for Hygiene and Public Health, University Hospital Bonn, Venusberg-Campus 1, Bonn, 53127, Germany

## Abstract

Microbial biofilms are complex communities embedded in a self-produced extracellular polymeric substance (EPS) matrix that can influence both decomposition and mineral formation on organic substrates. While biofilms are often associated with tissue degradation, they may also contribute to preservation processes through microbially mediated biomineralization.

In this study, we examined microbial community dynamics within leaf-associated biofilms and experimentally tested how selected metals and minerals influence the decay of biofilm-covered leaves under controlled laboratory conditions. Reconstructed microbial consortia derived from decaying leaves were exposed to varying ionic conditions to assess their effects on mineral precipitation and tissue degradation.

Biofilm-forming communities combined with elevated Ca^2+^ concentrations promoted mineral formation on leaf surfaces and significantly reduced degradation, as confirmed by chlorophyll retention measurements. The biofilm-forming capacity of representative isolates was validated using a crystal violet assay. Heavy metals produced variable effects depending on the microbial community present, highlighting the importance of metal-microbe interactions in decay processes. In addition, the iron-reducing bacterium *Shewanella* promoted the precipitation of diverse iron minerals on leaf substrates. These findings demonstrate that microbial biofilms can create localized microenvironments favourable for biomineralization and may contribute to early stages of leaf preservation in natural depositional environments.

## 1 Introduction

Biofilms, complex microbial communities encased within a self-produced extracellular polymeric matrix, are ubiquitous and play fundamental roles in various ecological processes [1,2]. Because of a multitude of niches within a biofilm, the microbial community can carry out various functions, such as decomposition and biomineralization [2].

Biomineralization is a process whereby microorganisms catalyze the precipitation of minerals, such as calcium carbonate, iron, silica, and phosphate minerals, within the EPS matrix [[3], [4], [5], [6], [7], [8]]. The minerals are deposited onto and within the biofilm substrate and contribute to the structural stability and functional diversity of the microbial community [9]. Microorganisms within biofilms can alter the composition of the solution to become supersaturated, favoring the precipitation of specific minerals [4]. Furthermore, metabolic activities like photosynthesis, sulfate reduction, denitrification, anaerobic sulfide oxidation, methanogenesis, and urea degradation can raise pH levels and facilitate mineral precipitation [3,7,10].

Additionally, the EPS matrix and the bacterial cell walls themselves can play a pivotal role in passive mineral precipitation through their negatively charged polymers, which bind cations and serve as nucleation points for mineral formation [11,12]. The synergy between microbial activities and the physicochemical properties of the EPS matrix and bacterial surfaces creates an environment conducive to biomineralization, resulting in the formation of a mineral matrix that reinforces and stabilizes plant tissues during preservation [13].

In contrast to the numerous studies on the involvement of biofilms in animal fossilization, experimental work on the preservation and subsequent fossilization of leaves is still emerging [[14], [15], [16], [17], [18], [19], [20], [21], [22], [23]]. Findings derived from experimental investigations indicate that the mineral enclosure of the leaf material that results through biomineralization may contribute to the preservation of plant cells by potentially hindering their degradation and disintegration, thereby enhancing their potential for fossilization [16,24,25].

Several studies on the early steps of fossilization have proposed a model for the preservation of soft tissues that suggests that biofilms in environments conducive to mineralization, with the addition of minerals, can slow down decay [8,25,26]. Such conditions are thought to arise shortly after burial, when oxygen becomes depleted and microbial metabolism creates geochemical microenvironments favourable for early diagenetic mineral formation. Fossil leaves are commonly carbonized, but they can also be permineralized with various minerals such as silica, calcium carbonate, pyrite, and iron oxides, allowing for exceptional three-dimensional preservation [[27], [28], [29], [30]]. Notably, iron oxides play a significant role in the preservation of leaves when precipitated externally in the presence of microbial biofilms [27]. Understanding the mechanisms underlying biomineralization within biofilms is therefore essential for elucidating the preservation pathways of plant remains, especially in plant soft tissues such as leaves and herbaceous stems, which are otherwise prone to rapid decay under natural conditions.

In this study, we experimentally investigated how microbial community composition and selected ionic conditions influence the decay and biomineralization of biofilm-covered leaves. *Hedera helix* was selected as a model system because its leaves host stable epiphytic and biofilm-associated bacterial communities and possess a thick, waxy cuticle that enables controlled multi-week decay experiments [21]. We focused on calcium, magnesium, iron, and selected heavy metals because of their reported roles in mineral precipitation and tissue preservation [[31], [32], [33], [34]]. In addition, the iron-reducing bacterium *Shewanella putrefaciens*, isolated from leaf biofilms, was included to examine its contribution to iron mineral formation. Based on previous studies, we tested three hypotheses: (1) microbial community composition influences plant tissue degradation; (2) EPS-rich biofilms promote ion capture and mineral nucleation; and (3) ionic conditions modulate biofilm structure and biomineralization. Together, these experiments were designed to test how microbial community composition and ionic conditions interact to influence biomineralization and the early preservation of plant tissues under controlled conditions.

## 2 Materials and methods

### 2.1 Sampling of water and plants and growth of biofilms *in vitro*

Water samples utilized in this study were obtained from the freshwater Tongrube Lake in Röttgen, located south of the city of Bonn in Germany (50°40′24.7″N/7°04′29.6″E). The water was collected in 1-L sterile glass bottles, and samples were refrigerated at 4°C during transportation to the laboratory. In the laboratory, the water samples were then filtered through sterilized coarse filters (grade 3hw; Binzer & Munktell Filter GmbH, Battenberg, Germany) to eliminate debris, sediment, and larger organisms to ensure sample purity and consistency.

Evergreen leaves of *Hedera helix* L. (common ivy) were collected from the vicinity of the Tongrube Lake near Bonn in Germany and were placed in sterilized glass petri dishes. A voucher specimen is deposited in the herbarium at the Nees Institute for the Biodiversity of Plants, Bonn Institute of Organismic Biology, University of Bonn, Germany.

For the isolation of the biofilm formers and analysis of the biofilm microbiomes, four leaves were then submerged in lake water in separate petri dishes. Three leaves were used for the DNA analysis, and one leaf for the isolation of biofilm strains. Biofilm growth was conducted under aerobic conditions at a constant temperature of 24°C in a darkened laboratory incubator for three weeks. Additional leaf samples were stored in anaerobic containers (BD GasPak™ EZ container) with freshwater treated with N_2_ gas for 20 min to establish anaerobic conditions using the BD GasPak™ system (BD BBL™ CO_2_ gas generators).

### 2.2 DNA sequencing

The microbial composition of the biofilms on *Hedera* leaves was analyzed after 1, 14, and 21 days. At each time point, three independent leaf samples (biological replicates) were removed from the pond water and transferred to a TE buffer for storage at −20°C. For the extraction of the DNA, the TE-buffered leaves were thawed and sonicated in an ultrasound bath (Branson 1210 Ultrasonic Cleaner; Emerson; St. Louis, Missouri, USA) for 2 min to remove the biofilms from the leaves. The detached biofilm microorganisms were collected on PORAFIL NC filters (0.25 μm, Macherey-Nagel GmbH & Co, KG, Germany) using vacuum filtration and stored at −20°C until DNA extraction. Total genomic DNA for each leaf was extracted using a Fast DNA spin kit for soil (MP Biomedicals) following the manufacturer's protocol. This test has been proven effective for DNA extraction of leaf biofilms [20]. The DNA was eluted in 50 μl Dnase/RNase-free water, and the concentration of the extracted nucleic acids was measured using a Qubit 4.0 fluorometer (Thermo Fisher Scientific, USA) with the dsDNA HS assay kit (Invitrogen).

The sequencing was performed by Biomarker Technologies (Beijing, China). To determine the prokaryotic composition in the leaf biofilms, we used the 16s-338 forward primer (5′-ACTCCTACGGGAGGCAGCA-3′) and the 16s-806R reverse primer (5′- GGACTACHVGGGTWTCTAAT-3′) to amplify the V3-V4 region of the 16S rRNA gene [18]. PCR amplification was performed with an initial denaturation at 95°C for 5 min, followed by 25 cycles of denaturation at 95°C for 30 s, annealing at 50°C for 30 s, and extension at 72°C for 40 s, followed by final elongation at 72°C for 7 min. The PCR product was purified with Agencourt AMPure XP Beads (Beckman Coulter, Indianapolis, IN) and quantified using the Qubit dsDNA HS Assay Kit and Qubit 4.0 Fluorometer (Invitrogen, Thermo Fisher Scientific, Oregon, USA). After individual quantification, the amplicons from each replicate were pooled in equimolar amounts for multiplexed sequencing, while maintaining replicate-level metadata for downstream analysis. The Illumina NovaSeq 6000 (Illumina, San Diego, CA, USA) was used for sequencing.

### 2.3 Sequence analysis

Raw sequences were processed using QIIME2 (version 2022.2) and DADA2 (version 1.16) [35,36]. This included filtering low-quality sequences, denoising, chimera filtering, and determining amplicon sequence variants (ASVs). Taxonomy was assigned using the Silva database (version 138) [37]. ASVs and taxonomy were then merged using R (version 4.21.1). We used the packages “ampvis2” (version 2.7.3) [38], “microeco” (version 0.20) [39], and “ggplot2” (version 3.4.2) [40] to analyze and visualize the microbial community composition alpha diversity.

### 2.4 Isolation of single bacterial strains from decaying leaves and reconstruction of communities

Using sterilized swabs, we removed the biofilm at each time point (day 1, day 14, and day 21). The biofilm was resuspended and incubated in a 96-well U-bottom plate with 200 μl tryptone soy broth (TSB) medium plus 1% glucose, and serial dilutions were prepared. The microtiter plate was then incubated for 72 h at 37°C under aerobic and anaerobic conditions. Subsequently, the diluted samples were streaked onto Columbia agar with 5% sheep blood (COL [BDTM, Becton, Dickinson and Company, New Jersey, USA]) and R-2A [Sigma-Aldrich, St. Louis, USA] followed by incubation for 24 h at 37°C. The plates with samples kept in anaerobic conditions were incubated in an anaerobic pot or anaerobic pouch system (BD Difco™ GasPak™ EZ Anaerobic System and Pouch systems, BD Becton).

Colonies were purified by repeated streaking until pure cultures were obtained, and pure isolates were identified using matrix-assisted laser desorption/ionization time-of-flight mass spectrometry (Microflex® LT MALDI-TOF/MS; Bruker Corporation, Billerica, USA) following the manufacturer's standard protocol for bacterial identification. Isolated strains were conserved as frozen stocks with 25% glycerol at −80°C. For all tests, the bacteria were grown at 30°C in their respective isolation medium and under aerobic and anaerobic conditions. Although initial biofilm development on leaves was performed at 24°C to reflect natural freshwater conditions, the use of 30-37°C during isolation and characterization follows standard microbiological protocols and facilitates broad-spectrum recovery of culturable taxa.

### 2.5 Reconstructed communities

The isolated strains were used in the biomineralization assays. We selected specific isolates based on their categorization in the literature as strong biofilm formers or plant decomposers [[41], [42], [43], [44], [45], [46], [47], [48], [49], [50], [51], [52], [53], [54], [55], [56], [57], [58], [59], [60], [61], [62], [63]]. These isolates were utilized individually (*Shewanella putrefaciens*) or in combinations as outlined in Table 1. Isolates were not selected to quantitatively mirror ASV relative abundances but to represent functional groups relevant to biofilm formation and leaf degradation. Accordingly, the experimental reconstruction aimed to capture functional traits associated with biofilm formation and decomposition rather than reproduce the exact natural community structure.

**Table 1:** List of strains showing reconstructed communities employed in the experimental biomineralization assays.

Bacterial isolate	Biofilm former	Decomposer	Mixed community
*Acidovorax defluvii* SK1	-	-	+
*Achromobacter spanius* SK2	-	+	+
*Acinetobacter pittii* SK3	+	-	+
*Aeromonas veronii* SK4	+	-	+
*Aquabacterium commune* SK5	-	-	+
*Azoarcus olearius* SK6	-	+	+
*Bacillus licheniformes* SK7	+	-	+
*Brevibacillus agri* SK8	-	+	+
*Citrobacter freundii* SK9	+	-	+
*Clostridium bifermentans* SK10	-	+	+
*Desulfovibrio desulfuricans* SK11	-	-	+
*Diskeya christantemi* SK12	-	+	+
*Erwinia rhapontici* SK13	-	+	+
*Hydrogenophaga pseudoflava* SK14	-	-	+
*Leifsonia soli* SK15	-	+	+
*Limnobacter thiooxidans* SK16	+	-	+
*Microbacterium phyllosphaerae* SK17	-	+	+
*Microvirgula aerodenitrificans* SK18	-	-	+
*Paenibacillus amylolyticus* SK19	-	+	+
*Paenibacillus glucanolyticus* SK20	-	+	+
*Pantoea agglomerans* SK21	+	-	+
*Pseudomonas fluorescens* SK22	+	-	+
*Pseudomonas putida* SK23	+	-	+
*Rhizobium radiobacter* SK24	+	-	+
*Shewanella putrefaciens* SK25	+	-	+

For the construction of the multispecies biofilm community (as outlined in Table 1), we selected 25 isolates. These selected bacterial strains were initially cultured in Müller-Hinton broth and incubated overnight at 37°C with continuous shaking at 200 rpm. Subsequently, cultures in the exponential growth phase, with an optical density (OD600) of 0.4-0.8, were used in the experiment. Subsequently, these cultures were centrifuged at 8000 rpm for 15 min at 4°C, washed with 1 × phosphate-buffered saline (PBS), and re-suspended in fresh Müller-Hinton broth. The optical density of each bacterial culture was adjusted to OD600 = 0.1. Following these preparations, all isolates were combined in equal volumes (100 μl) to initiate the next experimentation stage.

### 2.6 Biofilm formation assay

Biofilm-forming capacity of the selected bacterial strains within the “biofilm former” experimental assay was quantified using a crystal violet (CV) microtiter plate assay as described by O'Toole (2011) [64], with minor modifications. Briefly, strains were grown overnight in LB medium at 37°C with shaking (180 rpm), then diluted 1:100 into fresh LB medium. Aliquots (200 μL) were dispensed into flat-bottom 96-well polystyrene microplates (in triplicate) and incubated at 37°C for 24 h to allow biofilm formation.

After incubation, planktonic cells were removed by gently washing the wells with sterile phosphate-buffered saline (PBS) three times. The attached biofilms were stained with a 0.1% (w/v) crystal violet solution for 15 min. Excess stain was removed by rinsing wells with distilled water. The bound crystal violet was then solubilized in 95% ethanol, and absorbance was measured at 570 nm. A sterile medium served as a control in the biofilm formation assay.

Biofilm production was quantified as absorbance (OD_570_), and 95% ethanol was used as the blank control. Strains were categorized as weak, moderate, or strong biofilm producers based on OD_570_ values relative to the blank, following established criteria (Stepanović et al., 2000) [65].

### 2.7 Leaf disinfection and sterility assessment

For experiments involving reconstructed communities or *Shewanella putrefaciens* SK25, leaf fragments were thoroughly disinfected to remove surface-associated microbes. The protocol included immersion in 70% ethanol, followed by ultrasonic treatment (Branson 1210 Ultrasonic Cleaner; Emerson; St. Louis, Missouri, USA) for 2 min, and a final rinse in sterile water. Disinfected leaves were then transferred to sterile Falcon tubes containing autoclaved pond water for incubation under experimental conditions.

To assess the effectiveness of the disinfection protocol and evaluate potential contamination from residual endophytes or spore-forming bacteria, disinfected leaf fragments (uninoculated controls) were incubated in autoclaved pond water. After incubation, aliquots of the surrounding water and tissue surface were plated on nutrient agar and incubated at 37°C under aerobic and anaerobic conditions. Colony growth was observed and analyzed using MALDI-TOF mass spectrometry (Microflex® LT MALDI-TOF/MS Bruker Corporation, Billerica, USA).

### 2.8 Biomineralization assays

Individual strains were either inoculated individually or in various combinations as outlined in Table 1, categorized as “biofilm formers,” “decomposers,” or “mixed community.” A total of 150 μl of a single strain or multispecies biofilm community was added to 50 ml of autoclaved pond water for the biomineralization assays. The tubes were then incubated for 7 days to facilitate biofilm formation on the leaves at room temperature. Tubes were left open or closed as given in Table 2.

**Table 2:** List of the substances used in this study, which are known or suspected to enhance biomineralization. All experiments occurred over 7 days. The pH of the solutions was measured before leaf incubation. Assays with closed Falcon tubes limited gas exchange relative to open tubes and were therefore expected to provide lower oxygen availability during incubation. Dissolved oxygen concentrations were not directly measured.

List of substances	Concentration range	Storage	pH
**Ca^2+^**	10–100 mg/l	Falcon Tube, open lid	8.5–9
**Mg^2+^**	10–20 mg/l	Falcon Tube, open lid	8–8.5
**Ca^2+^ + Mg^2+^**	80 mg/l of Ca^2+^ + 20 mg/l of Mg^2+^	Falcon Tube, open lid	8–9
**CuS**	10–20 mM	Falcon Tube, closed lid	6.7–8
**ZnSO_4_**	10–20 mM	Falcon Tube, closed lid	7–7.5
**CdSO_4_**	10–20 mM	Falcon Tube, closed lid	6–7
**Al(H_2_O)_6_^3+^**	1–10 mM	Falcon Tube, closed lid	4–5
**Urea**	1–10 mM	Falcon Tube, closed lid	7–7.5
**FeSO_4_**	1 mM	Falcon Tube, closed lid	4–5

Triplicates of biofilm-coated leaves were thereafter individually transferred to separate Falcon tubes. To determine the minimum concentrations required to trigger biomineralization, each compound was tested across a range of concentrations, as detailed in Table 2. The concentration ranges tested in this study were selected based on previous experimental studies investigating biomineralization and soft-tissue preservation [[31], [32], [33], [34]], together with values reported from freshwater environments and geochemical studies where appropriate [66,67]. Various compounds known or suspected to enhance biomineralization were added to each tube, and all assays were performed in triplicate (9 leaves total per treatment). The tubes remained static throughout the incubation period and were not vortexed or otherwise agitated. The compounds tested and their respective concentration ranges are listed in Table 2.

Calcium and magnesium were additionally tested in combination because they are the predominant divalent cations in freshwater environments, where they naturally co-occur and collectively determine water hardness. Their combined effects were therefore examined to better approximate natural ionic conditions and to assess whether their coexistence influences biofilm-mediated biomineralization.

The same surface-disinfected leaves were used throughout all subsequent biomineralization assays. Following surface disinfection, leaves were inoculated with the respective experimental bacterial communities to allow biofilm formation prior to the mineralization experiments. Surface-disinfected control leaves were included in all experimental assays and incubated under the same experimental conditions, but without the addition of experimental bacterial communities. For simplicity, these samples are referred to as "no bacteria" throughout the manuscript and figure labels. However, this designation indicates the absence of deliberate bacterial inoculation rather than complete sterility, as residual endophytes or bacterial endospores may persist despite surface disinfection. Where appropriate, control leaves were also maintained without ion supplementation to provide a baseline for leaf degradation and potential mineral precipitation. Each compound was carefully measured and added within the concentration ranges shown in Table 2 to ensure controlled experimental conditions and to evaluate their effects on biomineralization potential. The biomineralization assays were carried out for one week. At the end of each experiment, leaves were removed for visual inspection and subsequent SEM analysis.

In addition, the percentage area of degraded leaf tissue was quantified using ImageJ (version 1.54) by segmenting leaf images with the Color Threshold tool (HSB colour space, Default thresholding method), followed by binary conversion and area measurement using the “Analyze Particles” function. The same thresholding workflow and parameter settings were applied consistently to all images to minimise user bias during image segmentation. This was performed on triplicate measurements from three biological replicates per treatment. Because the segmentation procedure produced several boundary values (0% and 100%) and minimal within-group variance, model assumptions for parametric or GLM-based approaches (e.g., binomial, quasibinomial, or beta-regression) were violated. As a result, we present degradation as descriptive statistics (mean ± standard deviation) and include full data visualization to reflect treatment differences. All statistical summaries were generated using GraphPad Prism 10.1.2.

### 2.9 Chlorophyll extraction and quantification

Fresh leaf fragments (∼50–250 mg) were incubated for 7 days under experimental conditions with selected microbial communities, including biofilm-forming isolates, decomposers, mixed communities, and sterile controls. Following incubation, chlorophyll was extracted from each sample in 1 mL of 96% ethanol by thorough grinding and centrifuged at 14,000 rpm for 5 min. The supernatant was collected, and absorbance was measured at 645 nm and 663 nm using a UV-Vis spectrophotometer (UviLine 9400, Schott, Mainz, Germany).

Chlorophyll *a and b* concentrations were calculated using Arnon's equations [68]:

• **Chlorophyll *a*** = 12.7 × A_663_ − 2.69 × A_645_
• **Chlorophyll *b*** = 22.9 × A_645_ − 4.68 × A_663_
• **Total chlorophyll** = Chlorophyll *a* + Chlorophyll *b*

Final chlorophyll values were normalized to the fresh weight of each leaf sample and expressed in mg chlorophyll per gram of fresh tissue. Chlorophyll content was analyzed using a one-way ANOVA with treatment as the fixed factor. Model assumptions were checked using residual diagnostics. Pairwise differences were tested using Tukey's HSD post-hoc test with family-wise confidence level of 95%.

### 2.10 Quantification of extracellular carbohydrate content

Extracellular carbohydrates associated with leaf biofilms were extracted from biofilm-covered leaves for subsequent determination of total carbohydrate content.For each treatment, biofilm-covered leaves were analyzed in triplicate, while freshly harvested leaves without biofilm served as controls. Approximately 0.5 g of leaf material with attached biofilm was placed in 50 mL glass jars containing 0.5 M H_2_SO_4_ and autoclaved at 121°C and 103 kPa for 60 min to extract extracellular polysaccharides. The supernatant was transferred to 50 mL Falcon tubes and stored at −20°C until analysis of total carbohydrate content.

Total extracellular carbohydrate content was determined using the phenol-sulfuric acid colorimetric assay. Briefly, 400 μL of the extracted supernatant was mixed with 10 μL of 80% phenol and 1 mL of 0.5 M sulfuric acid in Eppendorf tubes. Samples were incubated at room temperature for 10 min and subsequently at 30°C for 30 min to allow colour development. Absorbance was measured spectrophotometrically at 490 nm (UviLine 9400, Schott). Sucrose standards (1% and 10%) were used to generate calibration curves, and extracellular carbohydrate content concentrations were expressed as milligrams of sugar per gram of leaf material.

Differences in extracellular carbohydrate production among treatments were tested using one-way analysis of variance (ANOVA), followed by Tukey's honestly significant difference (HSD) test for pairwise comparisons. All analyses were performed in R (R10.2.6), and significance was evaluated at p < 0.05.

### 2.11 Scanning electron microscopy (SEM) with energy-dispersive X-ray spectrometry (EDS)

In preparation for scanning electron microscopy (SEM) analysis, small leaf sections were excised using a sterile scalpel. These sections were immediately immersed in a solution composed of 70% v/v ethanol and 4% v/v formaldehyde in water, and fixed for a minimum period of 24 h. Subsequently, the samples underwent a dehydration process employing graded ethanol solutions, culminating in immersion in 100% ethanol until critical point drying. The samples were dried with 22 cycles using a Leica EM CPDMA0 Critical Point Dryer at the Nees Institute for Biodiversity of Plants, Bonn Institute of Organismic Biology. The dried specimens were coated with a fine layer of palladium with a Cressington Sputter Coater 108 (Tesca GmbH, Germany). After coating, samples were mounted onto individual stubs using carbon tape and analyzed with a TESCAN Vega 4 SEM at the Division of Paleontology, Bonn Institute of Organismic Biology. Leaf observations were carried out utilizing the secondary electron (SE) and back-scattered electron (BSE) detectors in high-vacuum mode, with an acceleration voltage set at 20 kV. The minerals present on the leaves were further characterized using energy-dispersive X-ray spectroscopy (EDS). Three biological replicates per treatment were imaged, with five fields analyzed per sample. SEM–EDS spectra were used qualitatively to detect mineral precipitates on leaf surfaces. Attempts to perform quantitative comparisons were not feasible due to high intra-sample heterogeneity in mineral distribution and signal intensities, a well-recognized limitation of EDS-based analyses on biological samples.

## 3 Results

### 3.1 Biofilm formation on *Hedera helix* leaves *in vitro*

After one week of immersion in freshwater, the *Hedera* leaves developed very thin biofilms, and early signs of tissue degradation were observed. By the second week, a thick, slimy biofilm layer formed on the plant tissue, and by the third week, extensive decay of the leaf structure was observed (Fig. 1).

**Fig. 1:**
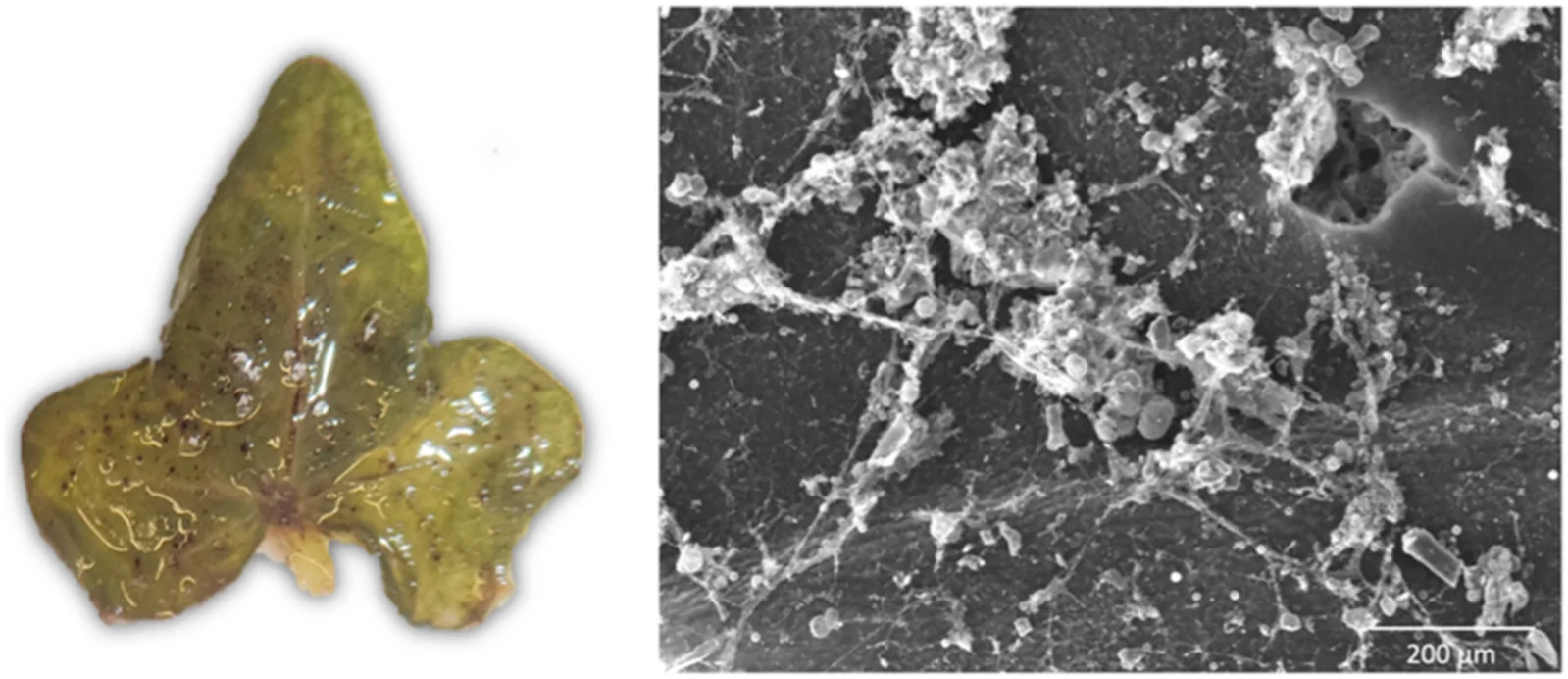
Development of microbial biofilms on *Hedera helix* leaves during *in vitro* incubation. Representative images of leaves incubated in natural pond water. Left: Macroscopic appearance of a leaf after two weeks of incubation showing the formation of a visible biofilm layer on the leaf surface. Right: Scanning electron micrograph (SEM) illustrating dense microbial colonization and biofilm formation on the leaf surface after two weeks of incubation. Scale bar: 200 μm.

### 3.2 Microbial biofilm community of *Hedera* leaves *in vitro* assays

In total, 1717 unique ASVs were detected across 9 samples after quality filtering. Sequencing depth ranged from 31,772 to 38,430 reads per sample, and the dataset was rarefied to 31,434 reads per sample for downstream diversity analyses.

Observed ASV richness showed a decreasing trend across time points (one-way ANOVA, p < 0.001). A set of 10 ASVs was detected across all time points (Fig. 2A; Additional file 1). Taxonomic analysis of the 16S rRNA ASVs revealed Proteobacteria (23.7%) to be the predominant phylum, followed by Firmicutes (19.2%), Bacteroidota (18.4%), Acidobacteriota (6.8%), and Verrucomicrobiota (5%). Relative abundance patterns suggested an increase in Firmicutes at day 14 and higher relative abundances of Desulfobacterota and Verrucomicrobiota at days 14-21 (Fig. 2B).

**Fig. 2:**
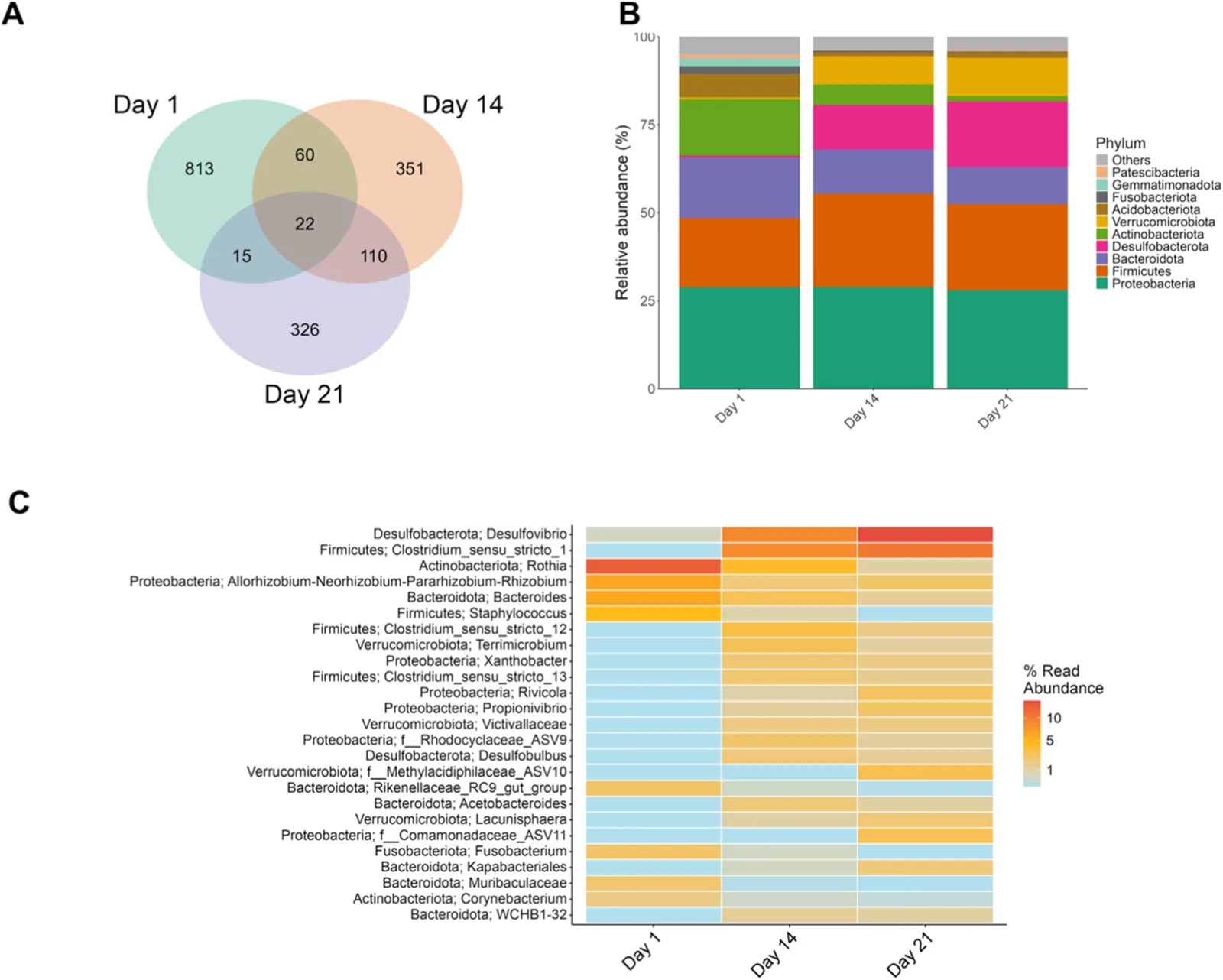
Temporal dynamics of bacterial biofilm communities on *Hedera helix* leaves determined by 16S rRNA gene amplicon sequencing. (A) Venn diagram showing the number of unique and shared amplicon sequence variants (ASVs) among biofilm samples collected after 7, 14, and 21 days of incubation. (B) Relative abundance of the dominant bacterial phyla across sampling times based on ASV composition. (C) Heatmap showing the relative abundance of the 25 most abundant bacterial genera identified in leaf biofilms. Colours represent relative abundance, with warmer colours indicating higher abundance. (For interpretation of the references to colour in this figure legend, the reader is referred to the Web version of this article.)

Temporal changes in relative abundance were evident among the top 25 bacterial genera. Among the most abundant genera, *Rothia*, *Rhizobium*, *Staphylococcus*, and members of *Kapabacteriales* decreased in relative abundance over time, whereas *Desulfovibrio* and *Clostridium* increased (Fig. 2C, Additional file 2). The effect of incubation time on community composition was confirmed by PERMANOVA (Bray-Curtis PERMANOVA: R^2^ = 0.77, F = 10.14, p = 0.001; Additional file 3.)

Several ASVs could not be confidently assigned to the genus level despite their high abundance in the biofilms. These ASVs included members of the families Rhodocyclaceae, Methylacidiphilaceae, Rikenellaceae, and Comamonadaceae. Notably, three distinct *Clostridium* ASVs, representing a genus commonly associated with anaerobic or oxygen-limited environments, appeared during the second week of the experiment. Although dissolved oxygen concentrations were not measured, this temporal shift is consistent with the expected reduction in oxygen availability within aging biofilms and decaying leaf tissues during prolonged incubation.

### 3.3 Isolation, functional characterization, and selection of bacterial strains for community reconstruction

A total of 71 different bacterial species were isolated from the biofilm samples collected from the *Hedera* leaves (Additional file 4). Forty-one isolates were obtained in the first week of sampling, followed by a greater number, 45 isolates, in the second week, and 46 isolates at the end of the experiment. In total, 43 genera were isolated from decaying leaves during the experiment.

Counts per sampling time include repeated taxa across time points; in total, 71 unique bacterial species (43 genera) were recovered across the entire experiment. The dominant genera among the isolated strains were *Pseudomonas* (8 strains), *Bacillus* (8 strains), and *Achromobacter* (5 strains), while *Aeromonas, Microbacterium*, *Leifosnia,* and *Citrobacter* were each represented by 3 strains.

A total of 10 isolated strains were selected to represent biofilm formers, while another set of 10 isolates was chosen to represent decomposers for the reconstructed community (Table 1).

To assess the biofilm-forming potential of selected isolates, we performed a Crystal Violet assay in 96-well plates. All ten selected biofilm formers exhibited measurable biofilm production, with varying intensities among isolates. Biofilm strength was categorized as weak, moderate, or strong based on established thresholds from the negative control, following the method of Stepanović et al. (2000) [65]. Three isolates (*Aeromonas veronii* SK4, *Pseudomonas fluorescens* SK22, and *Shewanella putrefaciens* SK25) demonstrated strong biofilm formation, while the rest were moderate or weak producers (Additional file 5). Crystal violet quantification provided a comparative measure of surface-attached biomass for the selected isolates. These measurements do not necessarily reflect biofilm behavior on leaf surfaces and are presented solely as phenotypic descriptors of individual strains, rather than as strict criteria for assigning functional roles, as the ability to form biofilms is widespread among bacteria.

Furthermore, several strains were chosen for their involvement in iron metabolism. Among the iron metabolizers were *Pseudomonas aeruginosa* [69], *Desulfovibrio desulfuricans* [70], and *Rhizobium radiobacter* [71]. Notably, *Shewanella putrefaciens* [72], a bacterium that can reduce iron and produce hydrogen disulfide, was employed specifically in iron mineralization assays. Thus, a total of 25 isolates were utilized in the construction of the reconstructed community (Table 1).

### 3.4 Leaf degradation in ion-supplemented microbial assays

Biomineralization assays *in vitro* were performed to ascertain the minimum concentration of calcium and magnesium ions required for leaf mineralization using both individual bacterial isolates and mixed reconstructed communities. All leaf samples with the biofilm formers showed fewer signs of decay (17%, Fig. 3A) compared to assays with reconstructed community (79.52%) and decomposers (91.83%). These assays were conducted in triplicate, and the percentage of decayed leaf area was quantified (Fig. 3B). Notably, the leaves that were stored in Falcon tubes with the biofilm formers and 80 mg/l of calcium exhibited the least amount of degradation (1.91%, Fig. 3B) and remained visually intact over the course of six weeks (Additional file 6).

**Fig. 3:**
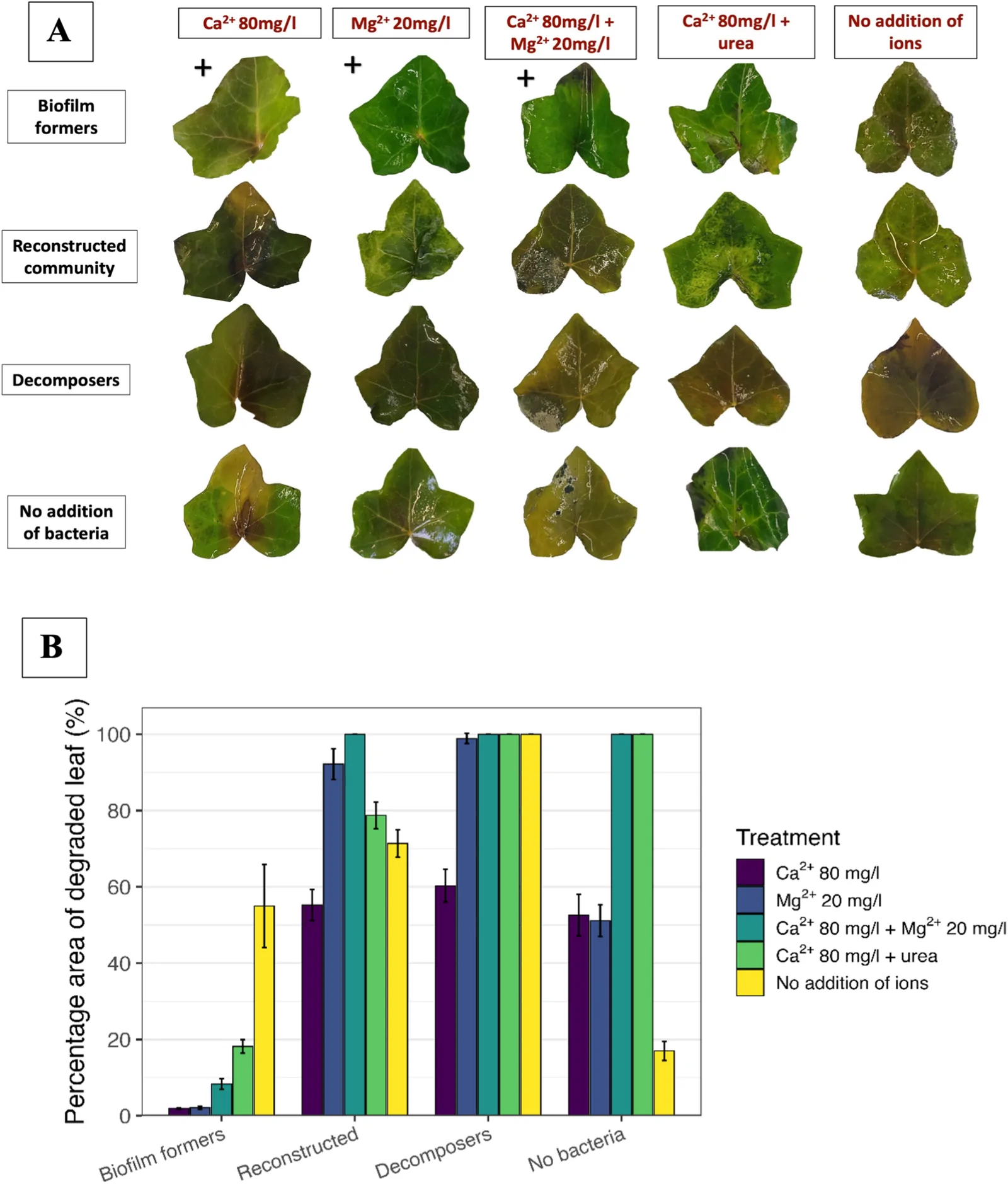
**A)** Photographic overview of decaying leaves of *Hedera helix* after one week of incubation in the presence of Ca^2+^, Mg^2+^, and urea. **B)** Histograms showing percentage of degraded leaf area after one week of experimental assays involving the addition of or in the presence of Ca^2+^, Mg^2+^, and urea. Degradation percentages were calculated from leaf triplicates using ImageJ. Leaves were inoculated with different mixtures of 25 strains isolated from decaying leaves previously and grouped according to their metabolism (Table 1). The column labelled “No addition of ions” represents leaves from assays where no further ions were added. Leaves with “no addition of bacteria” were sterilized; however, a further inoculation with the reconstructed community was omitted, that is, they show the decay caused by endophytic or spore-forming bacteria, such as clostridia or bacilli. The leaves marked by a plus sign are those where mineral precipitation was observed by SEM.

### 3.5 Chlorophyll retention as an indicator of tissue integrity

To confirm these visual observations, chlorophyll content was quantified as a biochemical marker for tissue integrity. Leaves treated with the biofilm-former community supplemented with calcium retained the highest levels of total chlorophyll, whereas those exposed to the decomposer community exhibited the lowest (Fig. 4). These results are consistent with hypothesis that biofilm-producing bacteria, particularly under calcium-enriched conditions, contribute to leaf tissue preservation. Because the post-incubation composition of the reconstructed communities was not re-sequenced, these outcomes reflect the emergent behaviour of the inoculated consortia rather than confirmed persistence of each strain. Although chlorophyll measurements were conducted at the community level, the observed trend suggests a potential link between stronger biofilm formation and delayed degradation. While slightly more degradation was observed in the other ion-supplemented treatments, the biofilm-former community consistently exhibited better visual preservation of leaf material compared to all other experimental groups. For Ca^2+^-supplemented assays, sufficient within-group variance allowed the application of one-way ANOVA, which showed a significant effect of treatment on degradation (p < 0.0001, Additional file 7). Tukey's HSD post-hoc comparisons further revealed that the biofilm-former community supplemented with Ca^2+^ differed significantly from all other treatments, exhibiting the lowest levels of degradation (all adjusted p < 0.0001).

**Fig. 4:**
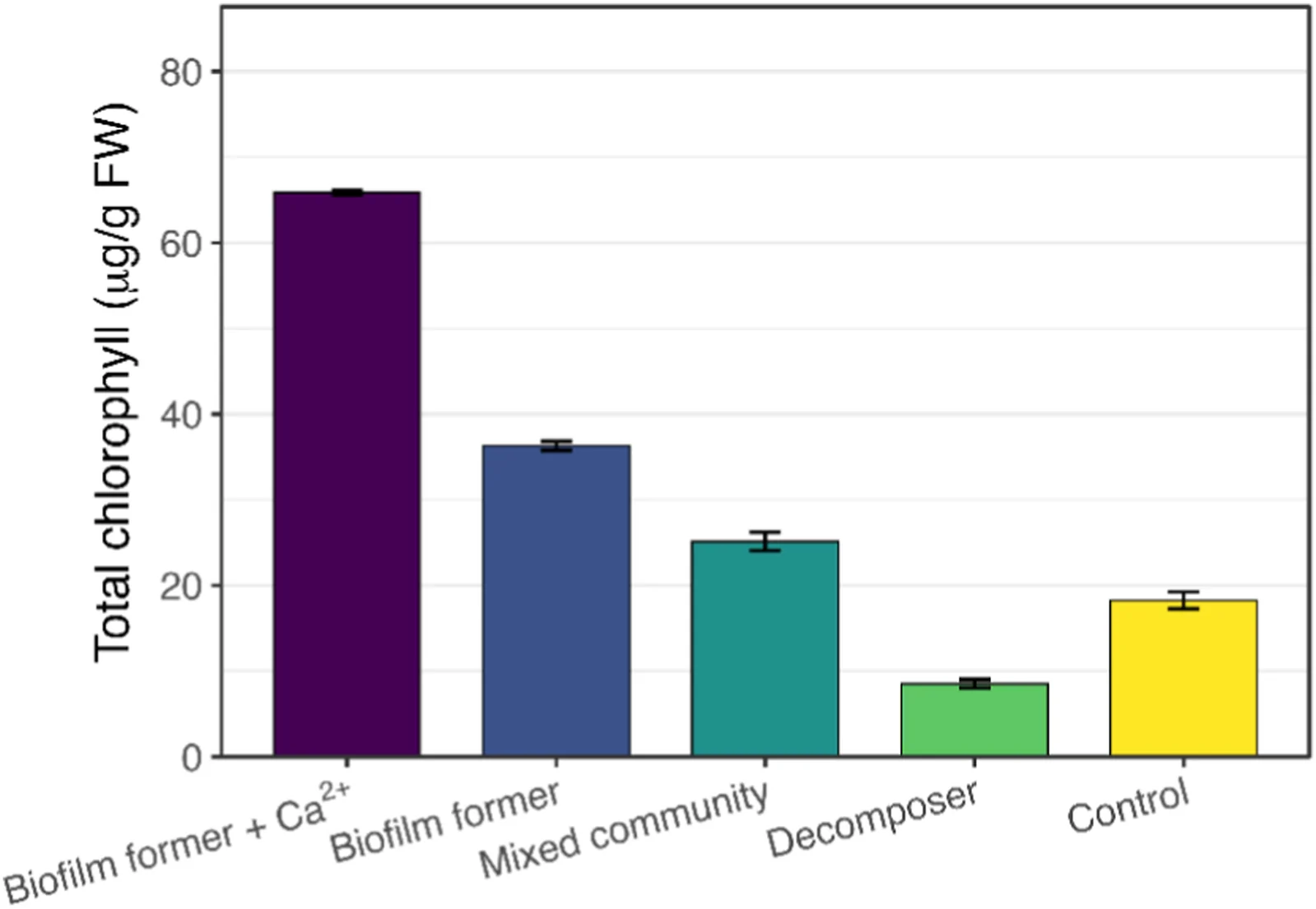
Mean chlorophyll content (chlorophyll *a* + b) (μg g^−1^ fresh weight) in *Hedera helix* leaves after 7 days of incubation with different microbial communities: biofilm formers supplemented with calcium, biofilm formers alone, a mixed community, decomposer strains, and an uninoculated control. Bars represent mean ± SD (n = 3), and numbers above the bars indicate mean values. A one-way ANOVA revealed a highly significant effect of treatment on chlorophyll retention (p < 0.0001). Pairwise comparisons using Tukey's HSD test showed that all treatments differed significantly (adjusted p < 0.05).

### 3.6 Extracellular carbohydrate content in microbial biofilms

To compare extracellular carbohydrate production among the different microbial treatments, extracellular carbohydrate content was quantified. Extracellular carbohydrate concentrations differed significantly among treatments (one-way ANOVA, p < 0.001). The highest extracellular carbohydrate levels were detected in the biofilm-former community supplemented with Ca^2+^ (Fig. 5). Post hoc Tukey comparisons showed that extracellular carbohydrate production in this treatment was significantly higher than in all other treatments, including the biofilm formers alone, the reconstructed mixed community, decomposers, and the uninoculated control (all adjusted p < 0.001). Intermediate extracellular carbohydrate concentrations were observed in the biofilm-former community without calcium and in the mixed microbial community, which did not differ significantly from each other. In contrast, extracellular carbohydrate levels in the decomposer treatment and the uninoculated control remained low and were not significantly different.

**Fig. 5:**
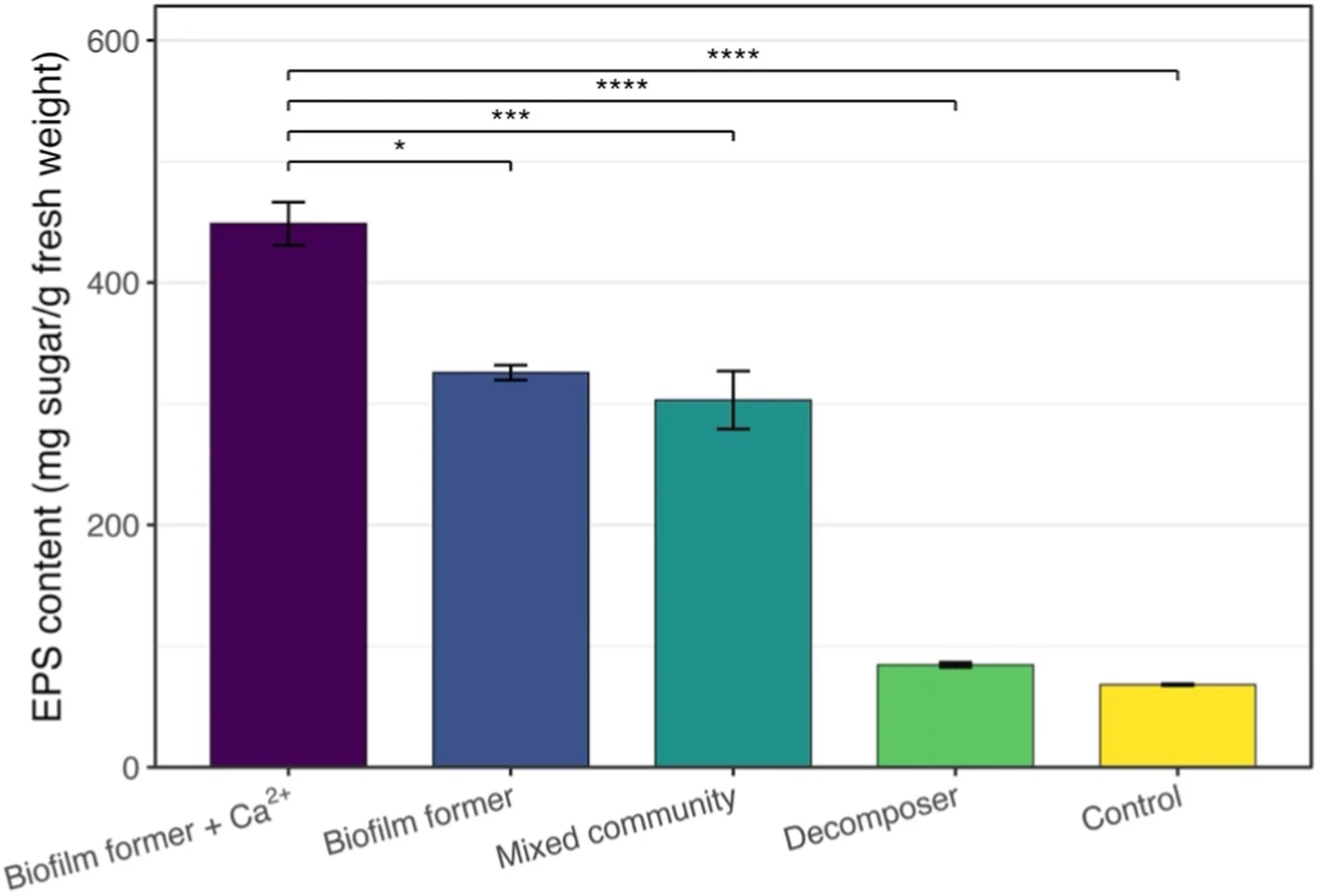
Extracellular carbohydrate content (mg sugar/g fresh weight) produced by microbial communities associated with *H. helix* leaves after 7 days of incubation. Treatments included biofilm formers supplemented with Ca^2+,^ biofilm formers alone, a mixed community, decomposer strains, and an uninoculated control. Bars represent mean ± SD (n = 3). Asterisks indicate statistically significant differences relative to the biofilm former + Ca^2+^ treatment (*p* < 0.05, **p < 0.001, ***p < 0.0001), based on one-way ANOVA followed by Tukey's HSD post-hoc test.

These results indicate that calcium supplementation was associated with increased extracellular carbohydrate production in the biofilm-forming community.

### 3.7 Biofilm-mediated mineral formation

Calcium minerals such as calcium carbonate-calcite (CaCO_3_) and calcium oxalate (CaC_2_O_4_), were observed exclusively on leaves with a calcium concentration of 80 mg/l or higher and inoculated with the biofilm former community. This was confirmed with SEM-EDS analysis (Fig. 6), even though oxalate druse crystals often formed in the mesophyll cells of leaves and in the cortex and pith cells of the *Hedera* stems [73].

**Fig. 6:**
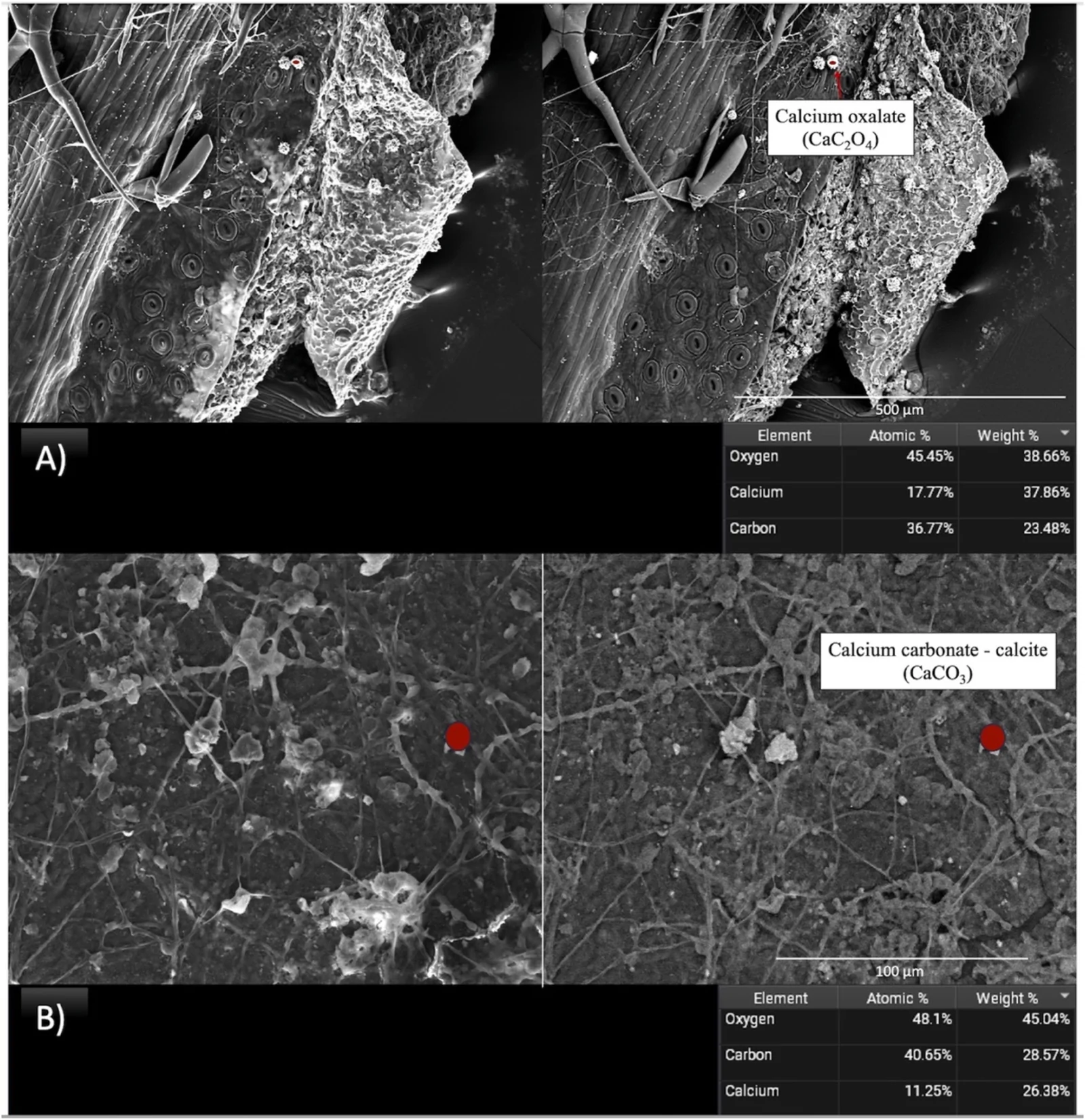
SEM–EDS images displaying calcium minerals formation in leaf biofilms in assays with a minimum calcium concentration of 80 mg/l and biofilm formers. A) Calcium oxalate (CaC_2_O_4_). B) Calcium carbonate–calcite (CaCO_3_). In both A and B, the left image was captured with the SE detector, while the right image was taken using the BSE detector.

Similarly, assays supplemented with a combined Ca^2+^-Mg^2+^treatment (80 mg/l Ca^2+^and 20 mg/l Mg^2+^) consistently resulted in the formation of calcium minerals. In contrast, leaves inoculated with the decomposers, the reconstructed community, or the controls (no inoculation), as well as those exposed to lower concentrations of calcium and magnesium, showed no signs of mineralization. SEM examination of the uninoculated control leaves revealed no mineral precipitates, indicating that mineral formation was associated with inoculated biofilm-forming communities under the tested ionic conditions.

### 3.8 Microbial contamination in uninoculated controls

Additionally, control leaves that had not been inoculated with reconstructed communities, but were stored in autoclaved pond water without added ions, still exhibited visible decay. This indicates that disinfection was incomplete, likely due to the presence of resilient endophytes which were not reached by the disinfectant or bacterial spores, that can only be killed by autoclaving or usage of aldehydes, both of which would destroy the integrity of the leaf tissue. Colonies recovered from these leaves were identified as *Bacillus* species by MALDI-TOF MS, supporting the hypothesis that endospore-forming bacteria contributed to degradation in uninoculated treatments. Fungal growth was also observed in some uninoculated controls, further suggesting that surface disinfection did not fully eliminate endogenous microbial contaminants.

### 3.9 The impact of heavy metal additions on leaf biofilms

Because heavy metals have been implicated in the fossilization of animal tissues, exploratory assays were conducted to evaluate whether selected metal ions influence microbial degradation of leaves. Zinc (Zn^2+^), copper (Cu^2+^), and cadmium (Cd^2+^) were introduced into experimental systems containing different microbial inocula under closed-lid conditions designed to limit oxygen diffusion and simulate early burial environments. Under such conditions, decreasing oxygen availability and pH may influence microbial activity and metal speciation [74]. Aluminium was additionally tested as Al(H_2_O)_6_^3+^ under acidic conditions (pH < 5), where this hydrated aluminium complex represents the dominant dissolved species [75].

Leaves exposed to heavy metals frequently developed chlorotic spotting, representing an early stress response that can precede tissue necrosis [76]. These visual symptoms were observed across several treatments but did not necessarily correspond to active tissue decay. Patterns of degradation largely reflected the composition of the microbial inoculum rather than the specific ions added (Supplementary File 8). Across treatments, leaves inoculated with biofilm-forming strains consistently showed lower degradation than those containing decomposer strains or reconstructed microbial communities. Average degradation in biofilm-former assays was approximately 35.97%, whereas reconstructed communities and decomposers exhibited substantially higher degradation (77.22% and 80.99%, respectively). Control leaves without bacterial inoculation also showed considerable decay (67.9%), likely reflecting the activity of endogenous microorganisms.

Within the biofilm-former treatments, the lowest degradation values were observed in assays supplemented with CuS (1.74%) and Al(H_2_O)_6_^3+^ (6.12%) at a concentration of 10 mM. However, statistical analysis did not detect significant differences across ion types or community treatments (one-way ANOVA, p = 0.77 and p = 0.09, respectively). Because several treatments produced boundary values (0% or 100% degradation) and limited within-group variance, these results are presented descriptively (mean ± SD).

Overall, the data indicate that biofilm microbial community composition had a stronger influence on leaf degradation than the presence of individual heavy metal ions. Ion effects were variable and appeared to depend on the inoculum rather than showing a consistent metal-specific pattern. Biofilm-forming communities showed mixed, ion-specific responses, whereas the reconstructed community displayed comparatively little change following ion addition. These observations suggest that individual strains within the consortia may respond differently to specific heavy metals, potentially reflecting the presence or absence of genetic determinants for metal resistance.

### 3.10 Mineralization assays with the addition of iron

In a separate experimental assay, we investigated the potential of the iron-reducing bacterium *Shewanella putrefaciens* to form biofilms and promote iron mineral precipitation on leaf substrates. *Shewanella* species are known to reduce ferric iron (Fe^3+^) to ferrous iron (Fe^2+^), thereby altering the redox state of iron and influencing its solubility, bioavailability, and precipitation dynamics [77]. SEM imaging combined with EDS analysis confirmed the presence of several iron minerals on the surfaces of leaves incubated with *Shewanella*, including iron carbonate (siderite, FeCO_3_), iron oxide (hematite, Fe_2_O_3_), and iron sulfide (pyrrhotite, Fe_1-x_S_x_) (Fig. 7).

**Fig. 7:**
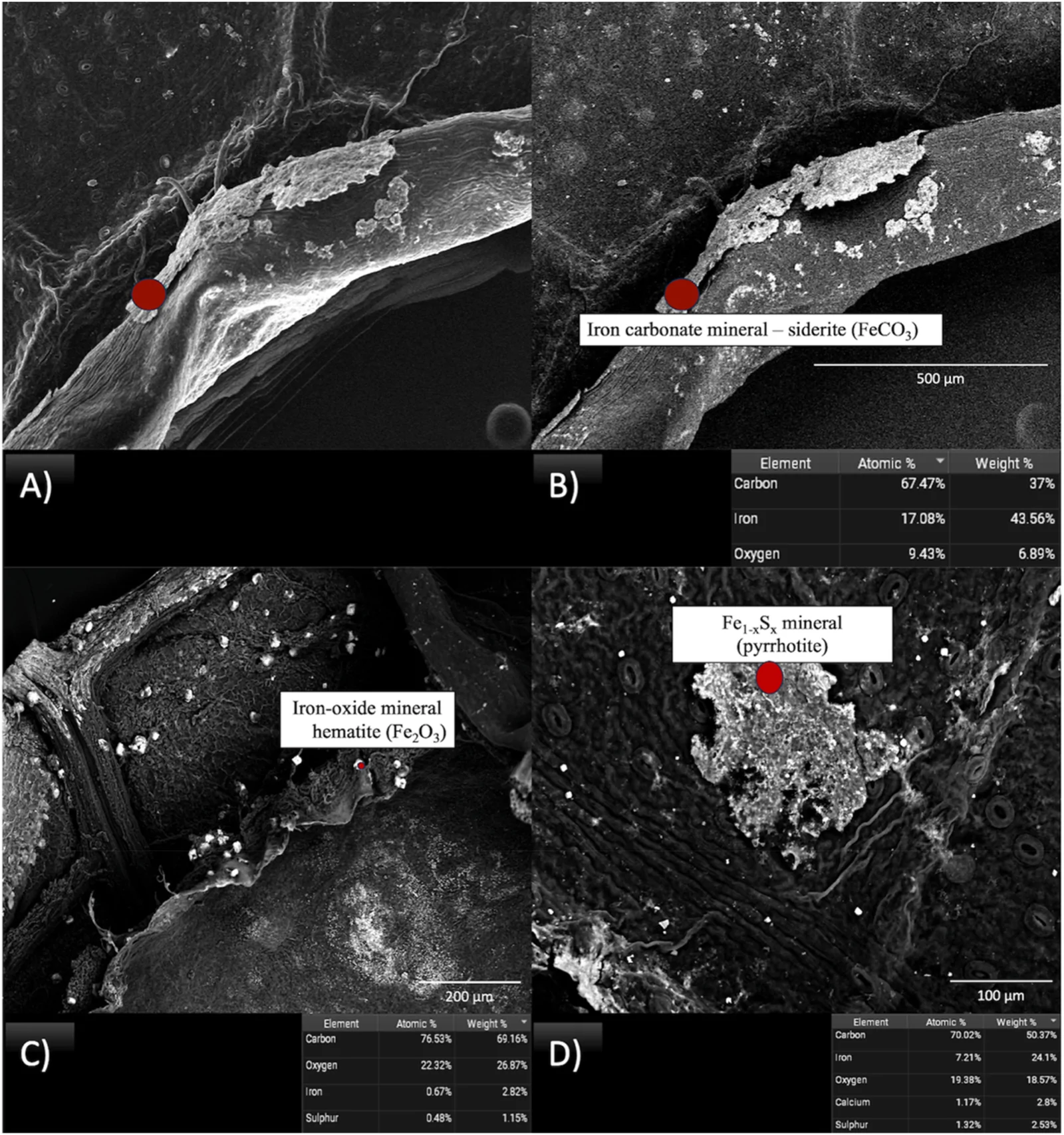
SEM–EDS analysis of iron minerals formed on *Hedera helix* leaves incubated with *Shewanella putrefaciens* and FeSO_4_. (A) Overview SEM image showing the mineralized leaf surface. (B) SEM image of the same mineralized region highlighting an iron carbonate precipitate identified as siderite (FeCO_3_) and selected for elemental analysis. (C) Representative iron oxide precipitate identified as hematite (Fe_2_O_3_). (D) Representative iron sulfide precipitate identified as pyrrhotite (Fe_1-x_S_x_). Mineral identification was based on SEM morphology together with EDS elemental composition. Scale bars are shown in each micrograph.

These mineral phases were consistently observed in association with bacterial biofilms on the leaf surfaces, suggesting that microbial iron reduction may facilitate localized iron mineral precipitation under the tested experimental conditions.

## 4 Discussion

Numerous studies have proposed that soft-tissue fossilization can involve low-oxygen conditions and mineral availability that promote surface mineralization [16,18,25,34,78,79]. The occurrence of microbes on fossil carbonized leaves and the complex microbial communities associated with leaf biofilms further suggest that biofilms may contribute to leaf preservation [15,18,21,22,[80], [81], [82]]. Here, we investigated how microbial community composition and selected ionic conditions influence leaf decay and biomineralization.

### 4.1 Microbial biofilms as dynamic communities during leaf decay

During the three-week decay experiment, microbial biofilms developing on leaf surfaces underwent pronounced shifts in community composition. Taxa such as *Rothia*, *Rhizobium*, *Staphylococcus*, and Kapabacteriales decreased in relative abundance, whereas *Desulfovibrio* and *Clostridium* increased. Similar genera have previously been reported in plant-associated biofilms studied under laboratory conditions [21].

Members of the sulfate-reducing genera *Desulfovibrio* and *Desulfobulbus* were relatively abundant in the leaf biofilms. *Desulfovibrio* species can utilize several electron acceptors, including sulfate, nitrate, and iron, and some strains can reduce toxic metals such as uranium(VI), chromium(VI), and iron(III) [83]. While typically anaerobic, certain strains do tolerate oxygen. In anoxic conditions, *Desulfovibrio* oxidizes organic matter, producing hydrogen sulfide, which reacts with iron to form iron sulfide and pyrite [84]. Pyritization is well known as an important pathway in the preservation of some plant fossils [25,[85], [86], [87], [88]].

The bacterium *Clostridium*, while widely distributed in soils and plants, has been less explored in terms of its colonization of plants, as indicated by the limited number of studies on this genus [21,89,90]. Notably, three distinct *Clostridium* ASVs (*Clostridium sensu stricto* 1, 12, and 13), representing a genus commonly associated with anaerobic or oxygen-limited environments, appeared during the second week of the experiment. Members of this genus are associated with soft-tissue decay and can also reduce iron and generate magnetite and iron sulfides [24,79,[91], [92], [93]]. Nitrogen-fixing genera such as *Rhizobium* and *Xanthobacter* were also abundant in the biofilms and may contribute to exopolysaccharide production and calcium carbonate precipitation [94,95].

Using cultivation-based approaches, we isolated and char acterized 71 bacterial strains from biofilm-coated decaying leaves. Representative isolates were selected based on taxonomic identity and reported functional traits associated with biofilm formation, organic matter degradation, or biomineralization to construct simplified experimental communities. These communities were designed to represent complementary functions rather than reproduce the relative abundances or structure of the natural community. The functional categories used in this study (‘biofilm formers’, ‘decomposers’, and ‘mixed community’) represent simplified experimental groupings rather than strict ecological classifications, as functional overlap between strains is expected. Accordingly, the crystal-violet assay should be interpreted as a descriptive measure of surface-attached biomass rather than evidence for exclusive biofilm-forming categories.

These findings indicate that leaf-associated biofilms host metabolically diverse communities capable of influencing both decomposition and mineral precipitation. However, fungal decomposers were not included, although fungal hyphae can contribute to early leaf decomposition and biofilm development in natural environments [21]. The present system should therefore be regarded as a simplified bacterial model of leaf decay and biomineralization.

### 4.2 Calcium-mediated biomineralization and leaf preservation

While it has been recognized that extant plants store calcium crystals in their cells [[96], [97], [98]], we focused on the formation of calcium crystals in biofilms on the surface of deteriorating leaves.

Calcium concentrations of 80 mg/l or higher promoted mineralization of decaying leaves in the presence of biofilm-forming communities under our experimental conditions. Calcium oxalate (CaC_2_O_4_) and calcium carbonate-calcite (CaCO_3_) formed on leaf surfaces, supporting the potential role of biofilm-mediated calcium mineralization in tissue preservation. The concentrations applied here were intended to delineate threshold conditions rather than reflect average environmental values. Although most freshwater systems contain calcium concentrations below 50 mg/l, values of 80–100 mg/l have been reported in natural settings [66,67].

Calcium biominerals are relatively scarce in the plant fossil record, likely because of their solubility and limited chemical stability [96,99]. Nevertheless, experimental studies have demonstrated that microbial biofilms can promote calcium carbonate precipitation and contribute to soft-tissue preservation [16,24,79,[100], [101], [102]].

Leaves inoculated with biofilm formers and supplemented with 80 mg/l calcium showed the lowest degradation after one week (1.91%, Fig. 3), and remained intact for over six weeks (Additional file 6). Uninoculated controls should be interpreted as surface-disinfected rather than completely sterile, because residual endophytes and endospore-forming bacteria persisted despite disinfection and may have contributed to the background degradation observed in these treatments.

The protective effect of the biofilm-forming community was further supported by chlorophyll retention, which was highest in leaves treated with biofilm formers supplemented with calcium (Fig. 4). Because chlorophyll degradation is an early indicator of tissue decay, this result is consistent with the reduced degradation observed in these treatments. Several isolates within this community, including *Pseudomonas fluorescens* and *Shewanella putrefaciens* also showed strong biofilm formation in the crystal-violet assay, although this *in vitro* phenotype does not necessarily reflect biofilm behaviour on leaf surfaces. Chlorophyll retention should likewise be interpreted as an indicator of short-term tissue integrity rather than long-term fossilization potential, which ultimately depends on the preservation of structural macromolecules such as lignin and cutin. The association between extracellular carbohydrate content, calcium mineral formation, and chlorophyll retention suggests that carbohydrate-rich biofilm matrices may contribute to leaf tissue stabilization.

Calcium oxalate crystals were also abundant on experimentally mineralized leaves. Although calcium oxalate is rarely preserved directly, crystal cavities interpreted as impressions of CaOx crystals occur in fossil leaves from different geological periods, including late Oligocene lake deposits in Germany [96].

Bacterial biofilms can promote calcium mineralization by trapping Ca^2+^ within the biofilm matrix and providing nucleation sites [7,12,25]. Microbial metabolism may further facilitate precipitation; for example, urease-mediated urea hydrolysis can increase pH and promote calcium carbonate formation [4,10,25,79,102]. However, our assays combining biofilm-forming strains, 80 mg/l calcium, and urea did not result in calcite or apatite formation, possibly because local pH conditions or urease activity were insufficient under the tested conditions.

### 4.3 Influence of heavy metals on microbial decay dynamics

We also explored whether heavy metals influence microbial leaf degradation in the experimental assays. Under acidic conditions (pH < 5), aluminium occurs predominantly as Al(H_2_O)_6_^3+^, a highly bioactive form known to affect microbial cells [75]. Both aluminium and copper can bind to bacterial cell envelopes and inhibit microbial activity [103]. However, many bacteria possess mechanisms that allow them to tolerate these metals, including the formation of Al^3+^–siderophore complexes or the presence of copper-resistance genes such as *copA* encoding multicopper oxidases [104,105].

Previous studies have suggested a role for metals in fossilization, including aluminium associated with permineralized bacterial microfossils [106,107]. In our experiments, aluminium and copper were associated with reduced degradation in some biofilm-former treatments, but these effects were not consistent across microbial communities. Such metal-resistance mechanisms may have evolved early and could have been further selected through interactions with protozoan predators that use metals to kill bacteria [108,109].

Overall, heavy-metal effects on leaf degradation were variable and appeared to depend more on microbial community composition than on metal exposure alone. Because no significant differences were detected among ion treatments, these observations should be interpreted cautiously. Nevertheless, the community-dependent responses suggest that metal–microbe interactions may contribute to heterogeneous patterns of tissue preservation under different geochemical conditions.

### 4.4 Iron biomineralization in leaf-associated biofilms

Unlike the reconstructed-community experiments, the iron assay specifically evaluated whether the leaf-associated isolate *Shewanella putrefaciens* could promote iron mineral precipitation in FeSO_4_-supplemented incubations.

Leaves incubated with *S. putrefaciens* and FeSO_4_ developed siderite, iron oxides, and Fe_(1-x)_S_x_ phases on their surfaces, as identified by SEM-EDS. *Shewanella* species are well known for enzymatic Fe^3+^ reduction, generating Fe^2+^ that can subsequently precipitate as insoluble iron minerals [110,111]. The association of these mineral phases with leaf biofilms therefore supports a microbial contribution to localized iron mineralization. Such mineral deposition may contribute to organic-matter stabilization and reduced decomposition [112].

Iron mineralization is an important pathway in plant preservation, with iron-encrusted leaves reported from several fossil deposits [17,18,27,111,[113], [114], [115]]. Iron oxides, oxyhydroxides, and carbonates can preserve fine leaf structures, and bacterial biofilms have been proposed to contribute to the formation of such mineral coatings [114].

Previous studies have demonstrated that *Shewanella* species can precipitate siderite and magnetite under specific geochemical conditions [72,[116], [117], [118], [119], [120], [121]]. Magnetite formation has been reported to prevail under N_2_-H_2_ atmospheres, whereas siderite formation can dominate under H_2_-CO_2_ conditions [116]. When *Shewanella* uses Fe^3+^(aq) as a terminal electron acceptor, the resulting Fe^2+^(aq) can, under suitable solution conditions, contribute to extracellular Fe_3_O_4_ precipitation [117,118], and biologically induced magnetite formation has been documented in several *Shewanella* species [72,[119], [120], [121]]. Rodríguez et al. (2004) also reported internal iron biomineralization in a perennial grass, further demonstrating the potential for plant-associated environments to support iron mineral formation [122].

The occurrence of iron oxides, carbonates, and sulphides within the same samples is consistent with microscale redox heterogeneity within biofilms. Localized oxygen consumption, diffusion limitation, and microbial metabolism can create adjacent oxidizing and reducing micro-niches, potentially allowing minerals associated with different geochemical conditions to precipitate within the same biofilm. However, dissolved oxygen concentrations were not measured during the experiments, and the oxygen conditions within individual incubation tubes therefore cannot be directly quantified. Open and closed tubes differed in their potential for gas exchange, but interpretations of oxygen availability and associated redox microenvironments remain inferential. Future experiments incorporating direct oxygen measurements would help clarify the contribution of oxygen gradients to biofilm-mediated mineral precipitation. In addition to the *Shewanella* strain used here, several isolates from the *Hedera* leaf microbiome have been reported to oxidize or reduce iron, suggesting broader biomineralization potential within this microbial community. To our knowledge, this study represents one of the first experimental demonstrations of iron mineral formation on decaying leaves *in vitro*. Because this study used *Hedera helix*, an evergreen species with a relatively thick cuticle, caution is warranted when extrapolating these findings to plant species with different leaf anatomies and decay dynamics. Future studies should therefore examine whether similar biofilm-mediated mineralization occurs in leaves with contrasting structural characteristics.

Taken together, our results demonstrate that microbial biofilms on decaying leaves can create localized geochemical microenvironments that promote mineral nucleation and reduce tissue degradation. Biofilm-forming communities combined with elevated calcium concentrations enhanced mineral precipitation and leaf preservation, whereas the effects of heavy metals varied with microbial community composition. In addition, the iron-reducing bacterium *Shewanella putrefaciens* promoted the precipitation of diverse iron minerals, highlighting the potential contribution of leaf-associated microorganisms to iron biomineralization. These findings support the view that the combined effects of biofilm formation, microbial metabolism, and ion availability represent an important but still underexplored mechanism contributing to the early stages of plant fossilization. Future studies should investigate biofilm-mediated biomineralization under natural depositional conditions and across plant species with contrasting leaf anatomies and microbial communities.

## CRediT authorship contribution statement

**Sabina Karačić:** Writing – original draft, Visualization, Methodology, Investigation, Formal analysis, Data curation, Conceptualization. **Brianne Palmer:** Writing – review & editing, Visualization, Formal analysis. **Jesenko Karačić:** Writing – review & editing, Investigation, Data curation. **Fabian Gäb:** Writing – review & editing, Investigation, Formal analysis. **Esther Sib:** Investigation, Data curation. **Carole T. Gee:** Writing – review & editing, Funding acquisition. **Gabriele Bierbaum:** Writing – review & editing, Funding acquisition, Conceptualization.

## Ethics approval and consent to participate

Not applicable.

## Consent for publication

Not applicable.

## Funding

This research has been funded by the Deutsche Forschungsgemeinschaft (DFG, German Research Foundation). This is contribution number 66 of the DFG Research Unit FOR 2685 “The Limits of the Fossil Record: Analytical and Experimental Approaches to Fossilization,” grant number 348043586 to G.B.and C.T.G., grant BI 504/15-16.

## Competing interests

The authors declare no competing interests.

## Data Availability

Raw sequence reads were deposited at the NCBI Sequence Read Archives under the BioProject accession number PRJNA1163075.
