# Supplementary material for: Microbial biofilms promote biomineralization and reduce leaf decay under controlled ionic conditions
Source: Biofilm. 2026 Oct 2;12:100403. doi: 10.1016/j.bioflm.2026.100403 (PMC13677424; doi:10.1016/j.bioflm.2026.100403)
Supplement: Supplementary file 1 [file mmc1.docx]

**Microbial biofilms promote biomineralization and reduce leaf decay under controlled ionic conditions**

**Additional Files**

**Additional file 1. List of core ASVs (10 ASVs)**

**Additional file 2. Relative abundance of the most abundant bacterial phyla over time.**
Stacked bar plots showing the relative abundance of bacterial phyla detected in *Hedera helix* leaf biofilms after 1 day (d1), 14 days (d14), and 21 days (d21) of incubation. Relative abundances were calculated from 16S rRNA gene amplicon sequencing data. Each bar represents an individual replicate sample.
Across all time points, the communities were dominated by Proteobacteria and Firmicutes, while other phyla contributed at lower relative abundances.

**Additional file 3. Principal coordinates analysis (PCoA) of bacterial community composition.** PCoA plot based on Bray-Curtis dissimilarity showing differences in bacterial community composition of Hedera helix leaf biofilms across incubation times (d1, d14, d21). Each point represents one replicate sample, and colours indicate sampling time.

**Additional file 4.** Bacterial species isolated from leaf biofilms. “Day 7” denotes isolates obtained after 7 days of *in vitro* leaf incubation, “Day 14” represents isolates harvested after 14 days, and “Day 21” signifies isolates collected after 21 days of *in vitro* leaf incubation.

| **Numer of isolate** | **Bacterial isolate** | **Day 7** | **Day 14** | **Day 21** |
| --- | --- | --- | --- | --- |
| 1 | *Acidovorax defluvii* | - | + | + |
| 2 | *Achromobacter mucicolens* | + | - | - |
| 3 | *Achromobacter insolitus* | - | - | + |
| 4 | *Achromobacter piechaudii* | - | + | - |
| 5 | *Achromobacter* xylosoxidans | + | + | + |
| 6 | *Achromobacter spanius* | - | + | + |
| 7 | *Acinetobacter pittii* | + | + | + |
| 8 | *Aeromonas ichthiosmia* | - | - | + |
| 9 | *Aeromonas popoffii* | + | + | + |
| 10 | *Aeromonas veronii* | + | + | + |
| 11 | *Aquabacterium commune* | + | - | - |
| 12 | *Azoarcus olearius* | + | - | - |
| 13 | *Azospira oryzae* | - | - | + |
| 14 | *Bacillus badius* | + | - | - |
| 15 | *Bacillus cereus* | + | + | + |
| 16 | *Bacillus firmus* | - | - | + |
| 17 | *Bacillus jeotgali* | - | + | + |
| 18 | *Bacillus licheniformes* | - | + | + |
| 19 | *Bacillus megaterium* | + | + | + |
| 20 | *Bacillus thuringiensis* | + | + | - |
| 21 | *Brevibacillus agri* | + | + | + |
| 22 | *Brevibacillus choshinensis* | + | + | + |
| 23 | *Buttiauxella gaviniae/brennerae* | + | + | + |
| 24 | *Citrobacter braakii* | - | + | - |
| 25 | *Citrobacter gillenii* | + | + | + |
| 26 | *Citrobacter freundii* | + | + | + |
| 27 | *Clostridium amylolyticum* | - | + | + |
| 28 | *Clostridium bifermentans* | - | - | + |
| 29 | *Desulfovibrio desulfuricans* | - | + | + |
| 30 | *Diskeya christantemi* | + | + | + |
| 31 | *Enterobacter cloacae* | + | - | - |
| 32 | *Escherichia coli* | + | - | - |
| 33 | *Escherichia marmotae* | + | - | - |
| 34 | *Exiguobacterium* sp. | + | + | + |
| 35 | *Erwinia rhapontici* | - | + | + |
| 36 | *Hydrogenophaga pseudoflava* | - | + | + |
| 37 | *Leifsonia aquatica* | + | - | - |
| 38 | *Leifsonia soli* | + | + | + |
| 39 | *Leifsonia shinshuensis* | - | + | - |
| 40 | *Lelliottia amnigena* | - | + | + |
| 41 | *Limnobacter thiooxidans* | + | + | + |
| 42 | *Lysinibacillus sphaericus* | + | + | + |
| 43 | *Microbacterium neimengense* | + | - | + |
| 44 | *Microbacterium testaceum* | + | + | + |
| 45 | *Microbacterium phyllosphaerae* | + | - | - |
| 46 | *Microvirgula aerodenitrificans* | - | - | + |
| 47 | *Niallia taxi* | - | + | + |
| 48 | *Paenibacillus amylolyticus* | - | + | + |
| 49 | *Paenibacillus glucanolyticus* | - | + | + |
| 50 | *Paenibacillus lautus* | - | + | - |
| 51 | *Pantoea agglomerans* | + | + | + |
| 52 | *Bacillus simplex* | + | - | - |
| 53 | *Pseudacidovorax intermedius* | - | + | + |
| 54 | *Pseudomonas agarici* | - | + | + |
| 55 | *Pseudomonas alcaligenes* | - | + | - |
| 56 | *Pseudomonas congelans* | - | - | + |
| 57 | *Pseudomonas fluorescens* | - | + | + |
| 58 | *Pseudomonas putida* | + | + | + |
| 59 | *Pseudomonas veronii* | + | + | + |
| 60 | *Pseudomonas vancouverensis* | + | - | - |
| 61 | *Pseudoxanthomonas mexicana* | - | + | + |
| 62 | *Raoultella ornithinolytica* | + | + | + |
| 63 | *Rhizobium radiobacter* | + | - | - |
| 64 | *Rhodococcus erythropolis* | + | - | - |
| 65 | *Romboutsia lituseburensis* | - | + | - |
| 66 | *Serratia marcescens* | + | + | - |
| 67 | *Shewanella putrefaciens* | - | - | + |
| 68 | *Sphingobacterium nematocida* | + | - | - |
| 69 | *Staphylococcus hominis* | + | - | - |
| 70 | *Stenotrophomonas sp.* | + | - | - |
| 71 | *Vogesella perlucida* | + | - | - |

**Additional file 5:** Biofilm formation of ten selected bacterial isolates assessed using the Crystal Violet assay (mean ± SD, n = 3). Based on Stepanović *et al*., 2000, isolates were classified as weak (≤ 0.504), moderate (0.504–1.008), or strong (> 1.008) biofilm formers.

**Additional file 6.** Photos of leaves with biofilms (above) and SEM images (lower left, SE detector, lower right, BSE) showing calcium carbonate structures on leaves, such as calcium roses, in assays with a minimum calcium concentration of 80 mg/l and the biofilm formers after 6 weeks on incubation.

**Additional file 7.** Statistical summary

**A) Effect of microbial community on leaf degradation with Ca/Mg assays**

(one-way ANOVA on % degraded area; N = 3 per treatment)

| **Comparison (Treatment groups)** | **Test** | **Statistic** | **p-value** | **Interpretation** |
| --- | --- | --- | --- | --- |
| Biofilm formers vs. Reconstructed vs. Decomposers vs. No bacteria | One-way ANOVA | F = 8.88 | **p = 0.0010** | Significant differences among treatments |
| Tukey’s HSD (pairwise) | — | — | **All adjusted p < 0.05** | Biofilm formers differ significantly from all other groups; least degradation |

**B) Effect of microbial community on total chlorophyll content**

(one-way ANOVA on chlorophyll concentration; N = 3 per treatment)

| **Comparison** | **Test** | **Statistic** | **p-value** | **Interpretation** |
| --- | --- | --- | --- | --- |
| Biofilm formers + Ca vs. Biofilm formers vs. Mixed vs. Decomposers vs. Control | One-way ANOVA | F = 8.98 | **p = 0.00015** | Strong treatment effect on chlorophyll preservation |
| Tukey’s HSD | — | — | **All pairwise comparisons p < 0.05** | Calcium + biofilm formers had significantly highest chlorophyll retention |

**C) Heavy metal assays** (ANOVA on % degraded area; N = 3 per treatment)

| **Comparison** | **Test** | **Statistic** | **p-value** | **Interpretation** |
| --- | --- | --- | --- | --- |
| Across metal treatments (Biofilm formers, Reconstructed, Decomposers, No bacteria) | One-way ANOVA | F = 0.49 | **p = 0.738** | No significant difference across ion types |
| Across community types (collapsed across ions) | One-way ANOVA | F = 2.55 | **p = 0.093** | Community differences not statistically significant |
| Notes | — | — | — | Strong trends observed, but variance & boundary values (0/100%) prevent significance |

**D) Mean percentage of degraded leaf area (± SD) across microbial inoculum treatments, averaged over all ion conditions.**

| **Treatment** | **Mean % degraded area (± SD)** |
| --- | --- |
| **Biofilm formers** | **35.97% ± 35.49%** |
| **Reconstructed community** | **77.22% ± 10.07%** |
| **Decomposers** | **80.99% ± 25.63%** |
| **No bacteria (controls)** | **54–68% depending on ion** |

**Additional file 8.** **The impact of heavy metal additions on leaf biofilms**

A) Photographic overview of decaying leaves of *Hedera helix* taken after one week of experimental assays involving the addition of heavy metals and aluminium. B) Histograms showing the percentage area in the degraded leaves taken after one week of experimental assays involving the addition of heavy metals and aluminium. Values represent descriptive means ± SD of proportional degradation scores derived from ImageJ segmentation (n = 3). Statistical modelling could not be applied due to boundary values (0% / 100%) and minimal within-group variance. Each percentage reflects the extent of degradation observed under each treatment condition. Leaves were inoculated with different mixtures of 25 bacterial strains, isolated from previously decaying leaves, and categorized based on their metabolic profiles (Table 1). “No addition of ions” refers to assays where no ions were added. “No bacteria” represents sterilized leaves without further inoculation, which display decay caused by endophytic or spore-forming bacteria such as clostridia and bacilli.

**Supplementary Data 1.**

**R script for microbiome data analysis and figure generation.**

Fully annotated R script containing the complete workflow for processing 16S rRNA amplicon sequencing data, taxonomic curation, diversity analyses, statistical analyses, and generation of publication figures.

# **Section 1: Initialization & Library Management**

Before starting execution, the graphics device is safely unlocked, and the workspace memory is cleared to prevent cross-contamination from previous sessions.

\# 1.1 Graphics device reset and workspace cleanup
while (!is.null(dev.list())) dev.off()
rm(list = ls())

\# 1.2 Load required packages
library(plyr) # Loaded first to prevent downstream select conflicts with dplyr
library(tidyverse)
library(data.table)
library(microeco)
library(phyloseq)
library(file2meco)
library(ggplot2)
library(ampvis2)
library(png)
library(grid)
library(gridExtra)

\# 1.3 Global publication typography standards
update_geom_defaults("text", list(family = "Arial"))
update_geom_defaults("label", list(family = "Arial"))

# **Section 2: Raw Data Processing & Quality Filtering**

This section ingests the raw operational taxonomic unit (OTU/ASV) count data directly from the BIOM conversion outputs, screens quality stats, and filters out global singletons to mitigate sequencing artifacts.

\# 2.1 Import raw feature (ASV) count table
count <- read.delim("/Users/briannepalmer/Desktop/Hedera/ASVs/feature-table.tsv",
 header = FALSE, row.names = NULL, stringsAsFactors = FALSE, na.strings = "n/a")

\# 2.2 Re-classify and re-format dataframe structure
colnames(count) <- as.character(unlist(count[2,]))
count1 <- count[-c(1,2),]
colnames(count1) <- "Feature.ID"

\# 2.3 Convert count classes to numeric variables
count2 <- count1
num_cols <- dim(count2)[2]
count2[2:num_cols] <- lapply(count2[2:num_cols], function(x) as.numeric(as.character(x)))

\# 2.4 Generate baseline dataset statistics for quality control
seq_total	<- apply(count2[2:num_cols], 2, sum) # Sequencing depth per sample
OTU_count	<- colSums(count2[2:num_cols] > 0)	# Observed richness per sample
OTU_single <- colSums(count2[2:num_cols] == 1)	# Singletons
OTU_double <- colSums(count2[2:num_cols] == 2)	# Doubletons
OTU_true	<- colSums(count2[2:num_cols] > 2)	# Confirmed ASVs (>2 counts)

dataset_info <- data.frame(seq_total, OTU_count, OTU_single, OTU_double, OTU_true)

\# 2.5 Quality Filtering: Remove global singletons
row_sums	<- apply(count2[2:num_cols], 1, sum)
count.no1	<- count2[row_sums > 1, ]

\# 2.6 Import taxonomy annotations and join with curated counts
taxname	<- read.delim("/Users/briannepalmer/Desktop/Hedera/ASVs/tax_dir/taxonomy.tsv",
 header = TRUE, row.names = NULL)
counts_wtax <- join(count.no1, taxname, by = "Feature.ID", type = "left", match = "first")

\# 2.7 Export high-quality annotated data table
write.csv(counts_wtax, file = "/Users/briannepalmer/Desktop/Hedera/OutputTable_wtax.csv",
 quote = FALSE, row.names = FALSE)

# **Section 3: Taxonomic Curation & Object Class Conversion**

Curated tables are loaded, non-target domains (Eukaryota) are removed, and the core ecosystem dataset is generated through Phyloseq into the microeco structure. Organelle pollution (chloroplasts/mitochondria) is strictly filtered out.

\# 3.1 Load curated dataset
data <- read.csv("/Users/karacic/Desktop/OutputTable_wtax.csv")

\# 3.2 Filter target bacterial and archaeal domains
data <- data %>% dplyr::filter(Kingdom %in% c("d__Bacteria", "d__Archaea"))

\# 3.3 Construct experimental metadata sheet
metadata <- data.frame(
 SampleName = c("Hedera_Day1", "Hedera_Day14", "Hedera_Day21"),
 Time = c("Day 1", "Day 14", "Day 21"),
 stringsAsFactors = FALSE
)

\# 3.4 Clean missing values and assign standard ASV designations
data[, c(2:4)][is.na(data[, c(2:4)])] <- 0
data$ASV <- as.vector(paste0("ASV", seq_len(nrow(data))))

\# 3.5 Format data objects for Phyloseq class conversion
otu.table	<- data.frame(data[, c(2:4)], row.names = data$ASV)
otu.table.ps <- otu_table(as.matrix(otu.table), taxa_are_rows = TRUE)

rownames(metadata) <- metadata$SampleName
sam.data.ps <- sample_data(metadata)

tax.table	<- data[, c(5:11)]
tax.table.ps <- tax_table(as.matrix(data.frame(tax.table, row.names = data$ASV)))

\# 3.6 Build unified Phyloseq object and transform into microeco class
ps <- phyloseq(otu.table.ps, tax.table.ps, sam.data.ps)
df <- phyloseq2meco(physeq = ps)

\# 3.7 Eliminate non-target organelle pollution (mitochondria & chloroplasts)
df$filter_pollution(taxa = c("mitochondria", "chloroplast"))
df$tidy_dataset()

# **Section 4: Abundance Profiling and Diversity Calculation**

Standard calculations are performed on the parsed community matrix to establish absolute indices for subsequent figures.

\# 4.1 Execute abundance profiles and diversity matrices
df$cal_abund()
df$cal_alphadiv(PD = FALSE)	# Alpha diversity indices
df$cal_betadiv(method = "bray") # Bray-Curtis beta distances

# **Section 5: Figure 2 Panel Components Generation**

Individual plots are customized to correct fonts, avoid label overlaps, and optimize widths before final multi-panel layout assembly.

setwd("/Users/karacic/Desktop/")

\# ---- PANEL A: VENN DIAGRAM (microeco) ----
t1_venn	<- trans_venn$new(df$merge_samples(group = "Time"), ratio = NULL)
p_venn_raw <- t1_venn$plot_venn(text_size = 7.5, text_name_size = 11)

p_venn_final <- p_venn_raw +
 theme(
 text = element_text(family = "Arial"),
 axis.text = element_blank(), axis.title = element_blank(),
 axis.ticks = element_blank(), axis.line = element_blank(),
 panel.grid = element_blank(),
 plot.margin = margin(t = 5, r = 5, b = 5, l = 5, unit = "pt")
 )
ggsave("hedera_venn_large.png", plot = p_venn_final, dpi = 300, width = 8, height = 7, units = "in")


\# ---- PANEL B: TAXONOMIC BARPLOT (microeco) ----
t1_bar <- trans_abund$new(dataset = df, taxrank = "Phylum", ntaxa = 10, groupmean = "Time")
g1_raw <- t1_bar$plot_bar(others_color = "grey70", legend_text_italic = FALSE)

g1_final <- g1_raw +
 theme_classic() +
 theme(
 text = element_text(family = "Arial"),
 axis.title.y	= element_text(size = 23),
 axis.text.x	= element_text(size = 19, angle = 45, hjust = 1, vjust = 1),
 axis.text.y	= element_text(size = 19),
 legend.title	= element_text(size = 21),
 legend.text	= element_text(size = 18),
 panel.grid.major = element_blank(), panel.grid.minor = element_blank(),
 panel.background = element_blank(), plot.background = element_rect(fill = "white"),
 strip.background = element_rect(fill = "white", color = "black"), strip.text = element_text(size = 18),
 legend.box.spacing = unit(0.2, "cm"),
 plot.margin	= margin(t = 5, r = 5, b = 5, l = 5, unit = "pt")
 )
ggsave("g1.png", plot = g1_final, dpi = 300, width = 12, height = 8, units = "in")


\# ---- PANEL C: RELATIVE ABUNDANCE HEATMAP (ampvis2) ----
d <- amp_load(otutable = df$otu_table, metadata = metadata, taxonomy = df$tax_table)
d.rare <- amp_rarefy(d, 31434)

heatmap_raw <- amp_heatmap(
 data = d.rare, group_by = "Time", tax_aggregate = "Genus", tax_add = "Phylum", tax_show = 25, plot_values = FALSE,
 color_vector = c("lightblue2", "darkgoldenrod1", "#E74C3C"), plot_colorscale = "sqrt", plot_legendbreaks = c(1, 5, 10)
)

theme_heatmap <- theme(
 text = element_text(family = "Arial"),
 axis.text.x	= element_text(angle = 45, size = 18, vjust = 1, hjust = 1),
 axis.text.y	= element_text(size = 14),
 legend.title	= element_text(size = 16), legend.text = element_text(size = 14),
 legend.position = "right",
 panel.background = element_blank(), panel.grid.major = element_blank(), panel.grid.minor = element_blank(),
 axis.line	= element_line(color = "black"), axis.ticks = element_line(color = "black"),
 legend.key	= element_rect(fill = "white", colour = "black"),
 plot.margin	= margin(t = 5, r = 5, b = 5, l = 20, unit = "pt")
)
heatmap_final <- heatmap_raw + theme_heatmap
ggsave("heatmap.png", plot = heatmap_final, dpi = 300, width = 14, height = 6.5, units = "in")

# **Section 6: Figure 2 Layout Composite Assembly**

The pre-rendered panels are merged using strict grid calculations to construct the final figure file without spatial text overlaps.

\# 6.1 Read high-resolution rasters
venn_img	<- readPNG("hedera_venn_large.png")
barplot_img <- readPNG("g1.png")
heatmap_img <- readPNG("heatmap.png")

venn_g	<- rasterGrob(venn_img, interpolate = TRUE)
barplot_g <- rasterGrob(barplot_img, interpolate = TRUE)
heatmap_g <- rasterGrob(heatmap_img, interpolate = TRUE)

\# 6.2 Set up publication tags (A, B, C) in matching Arial fonts
venn_panel <- arrangeGrob(
